# Sudden cardiac death in the adolescent population: a narrative review

**DOI:** 10.1186/s43162-023-00222-3

**Published:** 2023-05-19

**Authors:** Meet A. Patel, Anisha Malhotra, Franck H. Moussinga Mpondo, Vasu Gupta, Rahul Jain, Sachin Gupta, Rohit Jain

**Affiliations:** 1 Tianjin Medical University, Tianjin, People’s Republic of China; 2 Avalon University School of Medicine, Willemstad, Curaçao; 3 Dayanad Medical College & Hospital, Ludhiana, India; 4 Department of Internal Medicine, Penn State Milton S. Hershey Medical Center, Hershey, PA USA

**Keywords:** Sudden cardiac death, Cardiovascular disease, Cardiomyopathy

## Abstract

**Background:**

Death from unexpected circulatory arrest within 60 min of onset of symptom is known as sudden cardiac death (SCD). In spite of the advancement in treatment and prevention strategies, SCD remains the most common cause of death worldwide especially in the young.

**Main body:**

This review focuses on highlighting how different cardiovascular diseases contribute to SCD. We discuss the clinical symptoms that the patient experience prior to sudden cardiac arrest and the treatment strategies including pharmacological and surgical treatment.

**Conclusions:**

We conclude that since there are many causes of SCD and very few treatment options, prevention strategies, early detection, and resuscitation of those at greatest risk is important.

## Background

Sudden cardiac death remains a major problem worldwide and affects young healthy individuals and athletes. Hypertrophic cardiomyopathy is the leading cause of sudden cardiac death followed by anomalous origin of the coronary artery. This is mostly a leading issue in low- and middle-income countries where diagnosis is often delayed resulting in deaths of young individuals. In this review, we aim to highlight the pathophysiologic basis of SCD and clinical outcomes. In addition, the current strategies for prevention, identification, and resuscitation of those at greatest risk will also be discussed.

## Main text

Sudden cardiac death (SCD) is defined as natural death from unexpected circulatory arrest occurring within an hour of symptom onset [1]. SCD is one of the most common cause of death worldwide with higher frequency especially in the young [2]. According to the World Health Organization (WHO), cardiovascular disease (CVD) is the leading cause of death globally. An estimate of 17.9 million people died from CVD in 2019, accounting for 32% of all global deaths. Of these deaths, 85% were due to heart attack and stroke and at least three quarters of the world’s deaths from CVD occur in low- and middle-income countries. People living in these countries often do not have the benefit of primary health care programs for early detection and treatment of people with risk factors for CVD and those suffering from CVD have less access to effective health care services which respond to their needs. As a result, detection is often late in the course of the disease and people die at a younger age from CVD [3]. The trend analysis of Global Burden of Disease free online database on age-standardized CVD identified that the global mean trend of CVD incidence had an ascending trend until 1996 followed by a descending trend after this year. Nearly all the countries including developed and developing experienced an overall declining trend in CVD incidence and mortality rates from 1990 to 2017, while the survival rate of CVD patients during the same time period was rather stable. The incidence and mortality rates of CVD per 100,000 persons in developed countries was 901.8 and 373.9, respectively, in 1990, declined to 877.1 and 304.4, respectively, in 2017; demonstrating a decrease by about 14.4% and 23.8%, respectively, in this 28 year period. The incidence and mortality rates of CVD per 100,000 persons in developing countries were 1272.1 and 268.0, respectively, in 1990, decreased to 1088.8 and 204.1, respectively, in 2017, exhibiting that the decline in developing countries was only about 2.7% and 18.5%, respectively, during the same time period [4].

In young persons aged 1–35 years, most SCD are caused by potentially inherited heart diseases, including hypertrophic cardiomyopathy, arrhythmogenic cardiomyopathy, dilated cardiomyopathy, and primary arrhythmogenic disorders (e.g., congenital long QT syndrome and catecholaminergic polymorphic ventricular tachycardia). However, coronary artery disease, anomalous coronary arteries, congenital heart disease, and aortic dissection are also potential causes [5]. Myocarditis is another potential cause of SCD, but is often under-diagnosed. In developing countries, rheumatic carditis, trypanosomas cruzi, and bacterial infection such as diphtheria are the common cause of myocarditis; however, in the developed world, viral infection commonly causes myocarditis [6].

SCD in the young has conspicuous presentation, significantly affecting the individual’s family and community [7]. In developing countries, at household level, CVD contributed to poverty due to increased health expenditure and high-out of pocket cost. At the macro-economic level, CVD leads to economic hardships of low- and middle-income countries [3].

### Pathophysiology of sudden cardiac death

SCD is known to be the largest natural cause of death in the USA, estimating 180,000–300,000 deaths/year [8]. In developing countries, sudden cardiac death accounts for 6 million deaths per year, which is twice as much compared to developed countries [9].

**Fig. 1:**
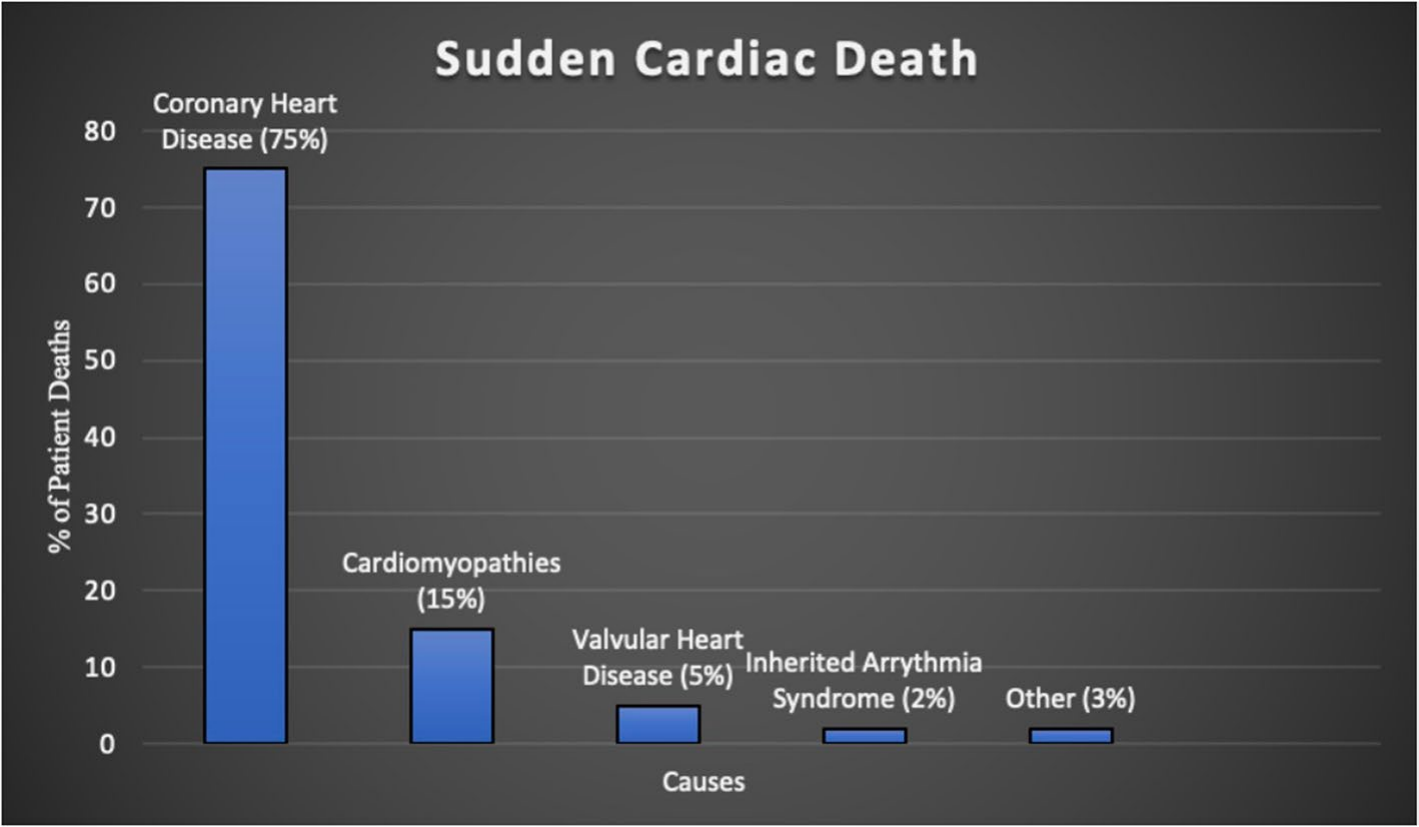
Causes of sudden cardiac death [8]

There are multiple causes that lead to sudden cardiac death, some of which are coronary heart disease (75%), cardiomyopathies (DCM, HCM, ARVC—15%), inherited arrhythmia syndromes (LQT, BS, CPVT, ERS—2%), cardiac abnormalities (genetic or acquired—3%), and valvular heart disease (5%) [8]. In young individuals < 35 years of age, HCM (36%) and ARVC/D (20%) are the two most common causes that lead to sudden cardiac death (Fig. 1).

#### Coronary artery disease (CAD)

Coronary artery disease (CAD) and atherosclerosis, it is known to be the major cause of SCD comprising 75% of patient deaths. One of the studies performed in Northern Finland between 1998 and 2017 with a total of 5869 subjects showed, “Most common underlying cause of SCD among these younger SCD victims (age 50 and younger) was CAD (43.6%)” [10]. CAD is caused by plaque buildup, acute occlusions, chronic obstructions, and ischemic cardiomyopathy. The factors that influence coronary heart disease causing SCD are individuals with ischemic burden, hemodynamic fluctuation, drugs, electrolytes, genetic factors, and autonomic variations [11].

Patients with a history of smoking, high or uncontrolled blood pressure, blood cholesterol levels, diabetes or insulin resistance, obesity, or overweight and genetic factors with past family history of heart disease are at high risk for CAD. The pathophysiology of SCD in CAD can be attributed to one of the following:

- A stable plaque transforming to an unstable plaque, causing transient ischemia, and potentially leadking to SCD or SCA.
- An acute coronary artery occlusion causing MI and leading to SCD.
- Chronic occlusion of vessel causing a scar formation which can further lead to the formation of arrhythmogenic circuits [11].

#### Cardiomyopathies

Cardiomyopathies are acquired or hereditary with autosomal dominant traits affecting the myocardium of the heart causing it to weaken its ability to pump blood to the body leading to sudden cardiac death. The three common types of cardiomyopathies that cause SCD are hypertrophic cardiomyopathy (HCM), dilated cardiomyopathy (DCM), and arrhythmogenic right ventricular cardiomyopathy/dysplasia (ARVC). There are 15% of patient deaths caused by cardiomyopathies leading to SCD in young individuals. There are risk factors that increases chance to acquire cardiomyopathy, some of which are long term high blood pressure, obesity, long-term alcohol and drug use (chemotherapy drugs), radiation, diabetes, thyroid abnormality, and other diseases like connective tissue disorder, amyloidosis, sarcoidosis, and hemochromatosis [12].

Hypertrophic cardiomyopathy accounts for 60% of patient deaths (out of 15%) causing SCD. HCM is caused by mutation in sarcomere protein gene causing the left ventricle wall to thicken, and the most common mutations are MYBPC3 and MYH7. The thickness of the left ventricle walls causes disruption of myocytes affecting signal conduction causing ventricular arrhythmias leading to sudden cardiac death [13].

Dilated cardiomyopathy occurs when there is myocyte damage or inflammation causing eccentric fibrosis of the myocardium leading to left ventricle chamber enlargement with decreased myocardial mass causing decrease in contractility of the heart. The decrease in contractility leads to compensation mechanism and negative remodeling of the heart, therefore dilating the left ventricle further. This causes further systolic dysfunction, hemostasis in ventricles, and thrombus formation risk leading to decreased cardiac output and volume overload, increasing the risk of cardiac arrhythmia causing sudden cardiac death [14].

**Fig. 2:**
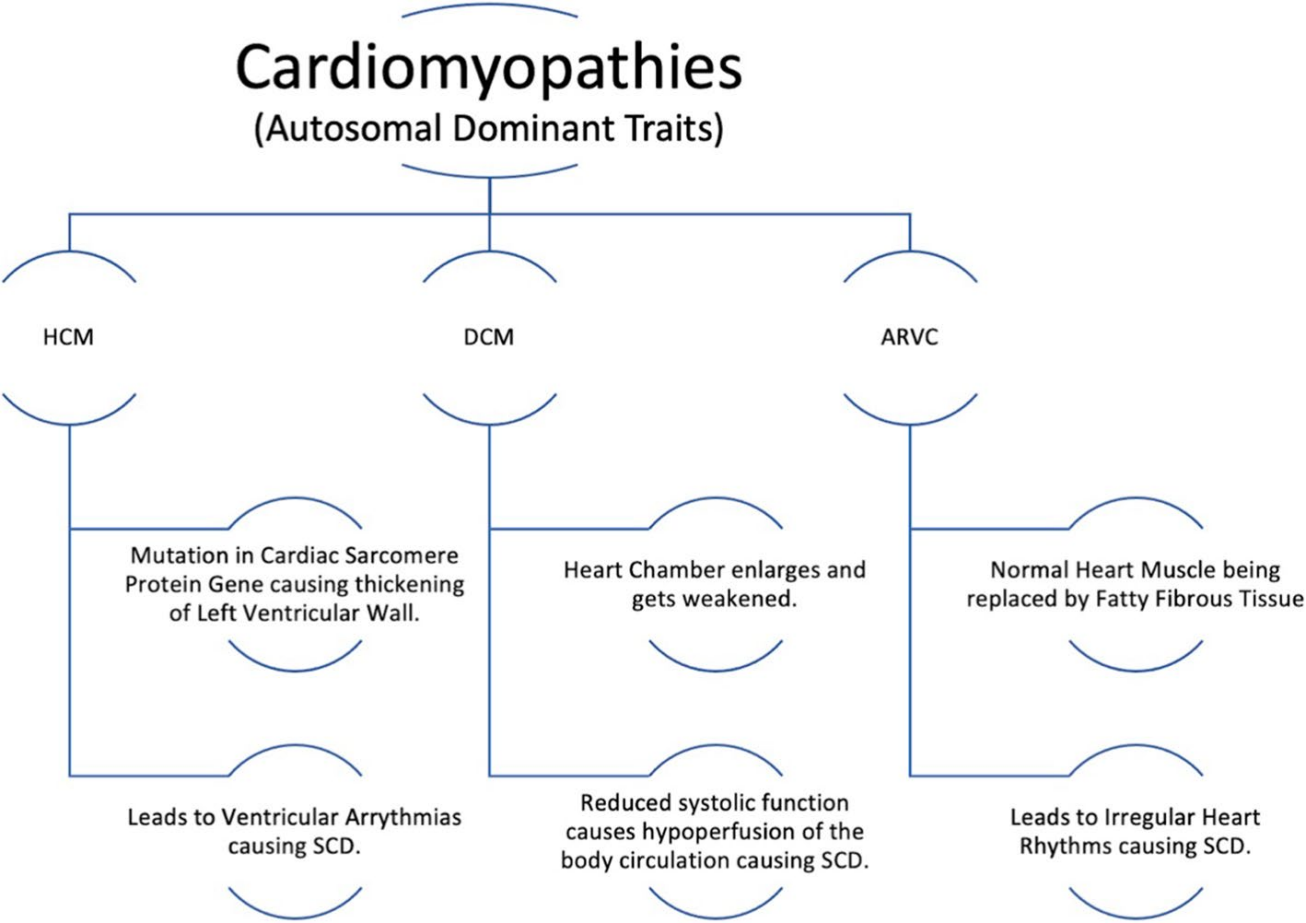
Types of cardiomyopathies causing SCD [13, 14, 17]. HCM, hypertrophic cardiomyopathy; DCM, dilated cardiomyopathy; ARCV, arrhythmogenic right ventricular cardiomyopathy

Arrhythmogenic right ventricular cardiomyopathy/dysplasia (ARVC/D) is an inherited myocardial disease that is associated with progressive right ventricular (RV) dysfunction caused by fibrous-fatty replacement of the myocardium of the heart. ARVC/D is the second most common cause leading to sudden cardiac death in young people. It is currently the second most common cause of SCD after hypertrophic cardiomyopathy in young people less than 35 years of age, causing up to 20% of deaths in this patient population. ARVC/D occurs due to desmosomal abnormalities which arise from mutations in cell adhesion proteins or intracellular signaling components [15]. The two main proteins whose reduced levels are associated with ARVC/D are desmoglein-2 and desmocollin-2, and their function is to maintain cell–cell adhesion and provide mechanical strength to tissue. These two desmosomal cadherins bind strongly with one another in the plasma membrane of the extracellular space and then bind to the linker proteins of the intracellular space. These linker proteins are plakoglobin, desmoplakin, and plakophilin-2. Plakoglobin and desmoplakin are responsible for anchoring desmosomes to desmin intermediate filaments [15]. Moreover, Plakoglobin also interlinks adherens junctions with the actin cytoskeleton and participates in cellular signaling. If there is defect in these linking sites of proteins, it causes disruption in cell to cell adhesion in increased mechanical stress leading to cell death and progressive loss of myocardium, which is then replaced by fibrous fatty tissue [16]. The remaining myocardial fibers within the fibrous fatty tissue zone causes slow conduction of heart leading to re-entry ventricular arrhythmias causing sudden cardiac death (Fig. 2).

#### Coronary artery anomalies (CAA)

Normal coronary artery anatomy is the one which can be found in more than 1% of population, and CAA can be defined as anomalous anatomy seen in less than 1% [18].

Anomalous coronary from opposite sinus (ACAOS) is a type of CAA which is most commonly associated with poor outcomes and prognosis in adolescent population [19]. ACAOS can further be classified as malignant or benign based on the course of the coronary artery. In the malignant subtype, the coronary artery travels between the aorta and pulmonary artery, known as the interarterial course, which is known to be associated with most cases of SCD found on the autopsies [20].

#### Inherited arrhythmia syndromes

##### Long QT syndrome (LQTS)

Long QT syndrome is dependent on duration of the ventricular action potential particularly QT interval which is influenced by closing and opening of ion channels. Sodium and calcium are positive ions whose influx results in depolarization of the heart, and potassium is a positive ion whose efflux causes repolarization. Excess of positive ions influx intracellularly causes prolongation of the ventricular action potential leading to long QT syndrome [21].

LQTS is classified in two categories, congenital and acquired. Congenital QT syndrome is caused due to mutation in genes that code for specific ion channel protein and their malfunction is what causes excess intracellular positivity leading to QT interval prolongation. There are 15 such genes, most common being KCNQ1 which causes LQT1 [21]. Long QT syndrome is usually triggered by emotional and/or physical stress. The mutant IKs does not sufficiently increase during β-adrenergic stimulation resulting in ventricular arrhythmias causing sudden cardiac death [22]. Acquired long QT syndrome is more commonly seen in individuals in comparison with congenital long QT syndrome. This is mainly due to disturbance of electrolytes, hypokalemia, hypocalcemia, and/or hypomagnesemia which leads to QT prolongation. There are also drugs that result in QT prolongation which block the outward IKr current encoded by KCNH2 gene, these drugs are Sotalol, Amiodarone, Macrolides, Fluoroquinolones, Haloperidol, Olanzapine, and Cisapride [21].

The notable subtypes of congenital LQTS are the Romano-Ward Syndrome (RW), Jervell and Lange-Nielsen (JLN) syndrome, and Timothy Syndrome (TS), and the later two of these are also known as malignant subtypes of LQTS. Studies have shown that JLN causes cardiac manifestations much early in life which does not respond to the treatment and TS, which is extremely rare, can present with a concomitant 2:1 atrio-ventricular block along with cognitive impairment, autism, and hypoglycemia [23].

##### Brugada Syndrome (BS)

BS is an electrical disease that is associated with mutation of gene SCN5A which encodes for human cardiac sodium channels on chromosome 3p21 required to work on Phase 0 of action potential. The mutation of this gene makes voltage gates cardiac sodium channel non-functional causing I(to) channel to stay unopposed in phase 1 of action potential causing transmural voltage gradient reentrant ventricular arrhythmias causing SCD [24].

Based on the ECG patterns, BS can be classified into two sub-types; type 1 with a coved ST segment and type 2, which has a saddleback ST segment. Type 1 is associated with most cases of SCD whereas type 2 is seen usually in stressful situations like fever, pneumonia, etc [25].

##### Catecholaminergic polymorphic ventricular tachycardia (CPVT)

**Fig. 3:**
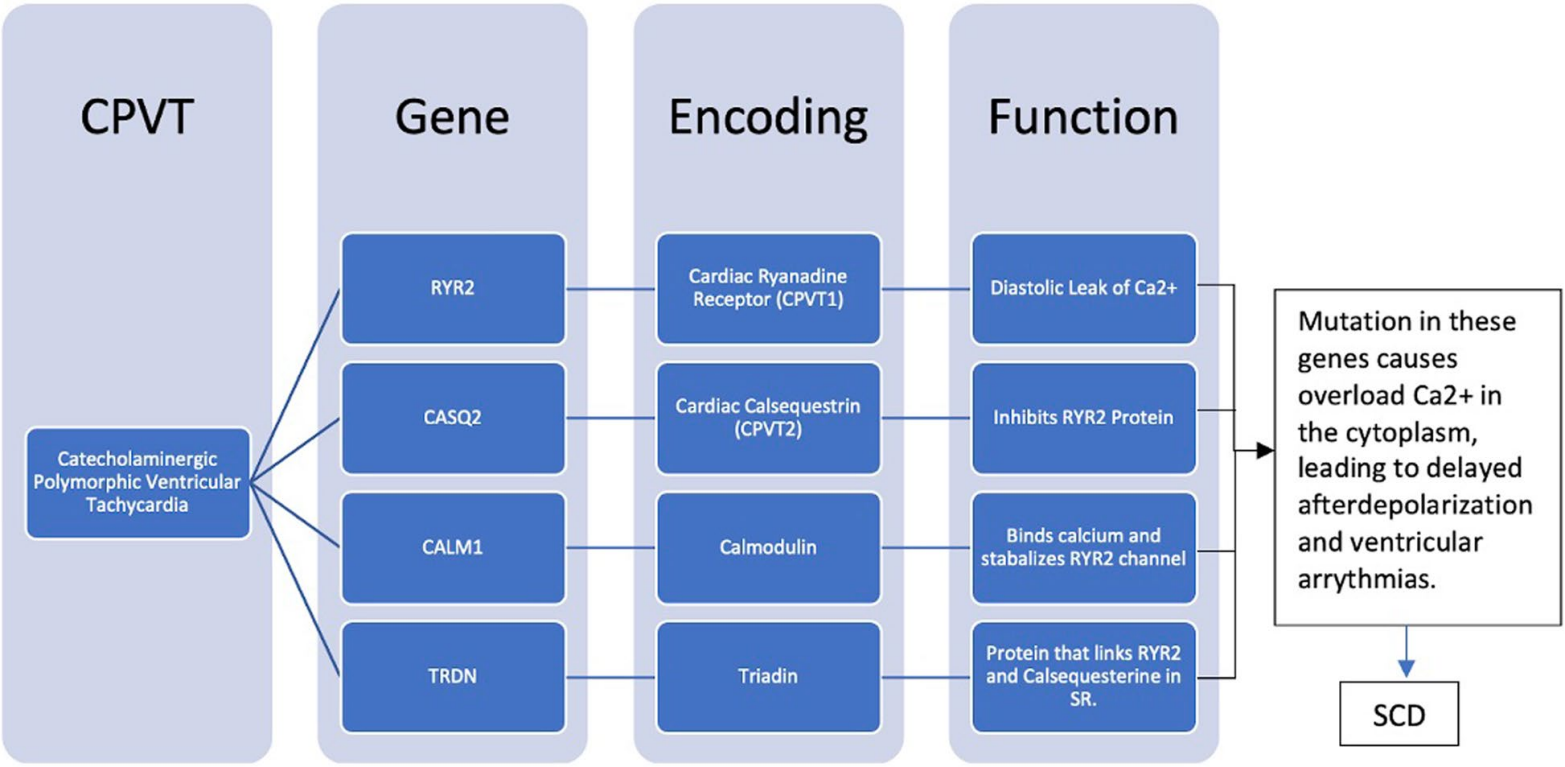
CPVT gene/protein mutations [22]. CPVT catecholaminergic polymorphic ventricular tachycardia

CPVT is caused by an increase in release of calcium from junctional sarcoplasmic reticulum (JSR) in the cytoplasm of cardiac myocytes. Calcium enters the cell through L-type Ca2 + voltage-gated channels which induces the RyR2 channel, releasing more calcium from the JSR into the cytoplasm following the Ca2 + -induced Ca2 + release mechanism [26]. The increase in intracellular Ca2 + induces sarcomere contraction by binding to troponin in the myofilaments, generating delayed afterdepolarization and ventricular arrhythmias leading to sudden cardiac death in some cases [27]. There are four main genes that are linked to CPVT which are RYR2, CASQ2, CALM1, and TRDN inducing Ca2 + release in the cardiac myocyte (Fig. 3).

On the basis of the clinical phenotype, it can be divided into typical and atypical CPVT. The pattern of arrhythmia is reproducible upon adrenergic activation (exercise) in typical but not in atypical CPVT [28].

##### Early repolarization syndrome (ERS)

Early repolarization syndrome occurs in the early phase of the cardiac action potential and is mainly caused by density changes of the cardiac transient outward potassium current/channel (Ito). Reduced density of Ito channels in the endocardium when compared to epicardium and/or mid-myocardium results in large Ito current influx in the early repolarization phase resulting in large voltage gradient generating J wave elevation [29]. This J wave elevation results in ventricular fibrillation initiating life-threatening arrhythmias leading to sudden cardiac death [25–30].  In patients with sudden cardiac death, previous standard 12-lead electrocardiogram on review would show J point elevation ≥ 1 mm in ≥ 2 continuous inferior and/or lateral leads [31].

#### COVID-19-associated myocarditis and its role in SCD

In patients with SARS-COV2 infection, the viral S protein interacts with the angiotensin-converting enzyme 2 (ACE2) on the surface of the host cell, inhibiting its action and causing myocarditis. Myocarditis has been found in 19% of COVID-19 hospitalized patients and is estimated to double in individuals with pre-existing cardiovascular and non-cardiovascular diseases, such as diabetes, hypertension, and cancers [32, 33]. These comorbidities may increase the likelihood of cardiac injury by making their cardiac myocytes more susceptible to attack by SARS-COV2. Among all hospitalized COVID-19 patients, the incidence of SCD was 1.8%, and in COVID-19 patients who passed away, the rate was 10%. This incident rate is significantly higher than that reported for patients without COVID-19 in the USA. The cause of this high SCD or cardiac arrest rate is currently not fully understood, and both cardiac (such as missed pulmonary embolisms) and non-cardiac (such as undetected ventricular fibrillation) factors may be responsible [33].

#### Commotio cordis

Commotio cordis is a rare form of SCD in young athletes. Its induction depends upon an object striking over the cardiac silhouette while it is susceptible, on upslope of *T* wave, during ventricular repolarization. The induction is further reliant on the velocity of the object striking the chest, and consequently, the energy of impact. The mortality rate of commotio cordis can be decreased by rapid recognition of the signs and symptoms of *commotio cordis*along with prompt implementation CPR and external defibrillation (if available) [34].

### Clinical symptoms and treatment

The patients prior to sudden cardiac arrest (SCA) may experience chest pain, palpitations, dizziness, shortness of breath, or near syncope. Although it is reported that almost half of the patients who have sudden cardiac arrest report no symptoms prior to collapse [35]. The American Heart Association has published guidelines for the pre-participation screening of high school and college athletes. This includes the athletes’ personal history, family history, and a physical exam [28–36]. Even though routine ECG is not recommended for the student athletes, those with personal history, family history, or abnormal physical exam should be referred for cardiac evaluation with ECG and echocardiogram [35].

The management of SCA is mainly focused on determining and treating the cause, maximizing the recovery of neurological and cardiac dysfunction [37].

The concept of primary prophylaxis by using implantable cardioverter-defibrillator (ICD) in children is far less studied than in adults. The benefits of using ICD in adults overweigh the risk of complications, which might not be true in children. However, physicians might follow the same algorithm for prophylaxis in children at a high risk of SCD as in adults [7].

The treatment modalities are mainly divided into pharmacological and surgical treatment.

#### Pharmacological treatment

The goal of pharmacological treatment is to increase the blood flow and decrease the risk of arrhythmias [38]. Only beta-blockers and amiodarone are found to reduce sudden death in myocardial infarction. Calcium channel antagonists, class I drugs (mexiletine, encainide, flecainide), and class III drugs (sotalol and dofetilide) all failed to reduce or even increased the incidence of sudden cardiac death after myocardial infarction [39].

#### Percutaneous treatment

Implantable cardioverter-defibrillator (ICD) are superior in reducing sudden death and total mortality compared with antiarrhythmic drugs therapy [29–37]. A meta-analysis of the antiarrhythmics versus implantable defibrillators (AVID), Cardiac Arrest Study Hamburg (CASH), and Canadian Implantable Defibrillator Study (CIDS) randomized trial of ICD vs pharmacological therapy in patients who have sustained ventricular fibrillation or ventricular tachycardia demonstrated 50% of relative risk reduction for arrhythmic death and 28% for all-cause mortality with ICD therapy [40]. Based on these findings, the first line treatment for survivor of SCD from ventricular tachycardia and ventricular fibrillation is ICD therapy [37].

##### Management for HOCM

Surgical septal myectomy is considered gold-standard treatment for HOCM. With confirmed mortality of less than 1% and clinical success rates of greater than 90 to 95%, techniques for HOCM treatment have evolved and allow gradient relief at any level of obstruction within the ventricle. A successful myectomy removes or lowers systolic anterior motion-mediated mitral regurgitation, as well as small amount of LV reverse remodeling and a reduction in size of the left atrium. Recurrent outflow tract obstruction is uncommon after surgical myectomy, and long-term survival is comparable to an age-matched general population. Similarly, procedures like alcohol septal ablation (ASA) have been improved to reach procedural mortality of < 1% at centers with skilled interventional teams. However, ASA may be less effective in patients with septal thickness ≥ 30 mm and resting gradient ≥ 100 mmHg. The greatest risk associated with ASA is conduction block requiring pacemaker and increased need for repeat intervention because of residual obstruction. Repeat ASA or myectomy is reported in 7 to 20% of patients after ASA [41].

Dual-chamber (DDD) pacing has been suggested as an alternative approach for the treatment of HOCM. Multiple studies have shown its effectiveness in reducing the left ventricular outflow tract (LVOT) gradient significantly at all time points including immediate, short term, and long term. However, DDD pacing does not reduce the LVOT gradient below 30 mmHg. Additionally, DDD pacing provides no significant reduction in septal thickness, and thus, it remains inferior to surgical myectomy in improving hemodynamic and functional measures. Moreover, it does not significantly reduce septal thickness, making it less effective than ASA in that aspect [42].

## Conclusions

SCD is a leading cause of mortality in the USA and worldwide, with the majority of cases attributed to inherited cardiovascular diseases (CVDs). According to the World Health Organization (WHO), CVDs were responsible for 32% of global death in 2019. Low- and middle-income countries are disproportionately affected, with SCD-related deaths often having significant impacts on families and communities. Therefore, understanding the pathophysiological basis of SCD and its clinical outcomes is critical. Coronary artery disease accounts for 75% of SCD deaths in people under the age of 50, while cardiomyopathies, both acquired and inherited, can also lead to SCD. The three common types of cardiomyopathies associated with SCD are hypertrophic cardiomyopathy, dilated cardiomyopathy, and arrhythmogenic right ventricular cardiomyopathy. Inherited arrhythmia syndromes, such as long QT syndrome, Brugada Syndrome, and early repolarization syndrome, are also leading causes of SCD. Treatment options for SCD are limited to pharmacological and surgical interventions. Therefore, it is important to focus on prevention strategies, early identification, and resuscitation of individuals at high risk.

## Data Availability

Not applicable.

## Abbreviations

SCD: Sudden cardiac death
WHO: World Health Organization
CVD: Cardiovascular disease
CAD: Coronary artery disease
HCM: Hypertrophic cardiomyopathy
DCM: Dilated cardiomyopathy
ARVC/D: Arrhythmogenic right ventricular cardiomyopathy/dysplasia
LQTS: Long QT syndrome
BS: Brugada syndrome
CPVT: Catecholaminergic polymorphic ventricular tachycardia
ERS: Early repolarization syndrome
ICD: Implantable cardioverter-defibrillator
